# Classical swine fever virus triggers RIG-I and MDA5-dependent signaling pathway to IRF-3 and NF-κB activation to promote secretion of interferon and inflammatory cytokines in porcine alveolar macrophages

**DOI:** 10.1186/1743-422X-10-286

**Published:** 2013-09-13

**Authors:** Xiao-Ying Dong, Wen-Jun Liu, Ming-Qiu Zhao, Jia-Ying Wang, Jing-Jing Pei, Yong-Wen Luo, Chun-Mei Ju, Jin-Ding Chen

**Affiliations:** 1 College of Veterinary Medicine, South China Agricultural University, No.483 Wu Shan Road, Tian He District, Guangzhou 510642, China; 2 College of Yingdong Agricultural Science and Engineering, Shaoguan University, Daxue Avenue, Zhenjiang District, Shaoguan 512005, China

**Keywords:** RIG-I, MDA5, CSF, CSFV, IRF-3, NF-κB, Interferon, Inflammatory response, PAM

## Abstract

**Background:**

Classical swine fever (CSF) caused by CSF virus (CSFV) is a highly contagious disease of pigs. The RNA helicases retinoic acid-inducible gene I (RIG-I) and melanoma differentiation-associated gene 5 (MDA-5) are differentially involved in the detection of various RNA viruses. In present study, we investigated the roles of RIG-I and MDA-5 in eliciting antiviral and inflammatory responses to CSFV shimen strain in Porcine alveolar macrophages (PAMs).

**Methods:**

CSFV Shimen strain was used as challenge virus in this study and PAMs were cultured *in vitro*. Interferon regulatory factor (IRF)-3 and nuclear factor-kappa B (NF-κB) translocation was detected using immunofluorescent staining; RIG-I, MDA5, interferon promoter-stimulating factor 1 (IPS-1), IRF-3 and NF-κB expression was measured by Western Blotting; Interferon beta (IFN-β), IFN-α, interleukin-1beta (IL-1β), IL-6 and tumor necrosis factor (TNF-α) expression was tested by Enzyme-linked immunosorbent assays (ELISA) and shRNA-mediated knockdown of MDA5 or RIG-I was performed.

**Results:**

The findings suggested that the initial response to CSFV infection resulted in the higher expression of RIG-I and MDA5 leading to the activation of IPS-1, IRF-3 and NF-κB in a dose-dependent manner. Evaluation of IFN-α, IFN-β, IL-1β, IL-6 or TNF-α expressed by PAMs showed significant differences between infected and uninfected cells. CSFV infected cells induced to express high levels of IFN-α, IFN-β, IL-1β, IL-6 and TNF-α in a dose-dependent way within 24 h post-infection (hpi). At the same time, CSFV improved the nuclear translocation of IRF-3 and NF-κB. We also directly compared and assessed the roles of RIG-I and MDA5 in triggering innate immune actions during CSFV infection through shRNA-mediated knockdown of MDA5 or RIG-I. We found that, compared to the control, the production of IFN-α, IFN-β, IL-1β, IL-6 and TNF-α in response to CSFV infection was heavily reduced in RIG-I knockdown cells while it was moderately decreased in MDA5 knockdown cells. PAMs derived from knockdown of both RIG-I and MDA5 almost failed to produce IFNs and inflammatory cytokines.

**Conclusions:**

It indicates that CSFV can be recognized by both RIG-I and MDA5 to initiate the RIG-I signaling pathway to trigger innate defenses against infection.

## Background

Classical swine fever (CSF) caused by CSF virus (CSFV), is a highly contagious disease of swine that is characterized by fever, hemorrhage, leukopenia, abortion, and high mortality [1]. Acute and chronic forms of CSF can be distinguished based on virulence and host range phenotype. Infection with highly virulent CSFV strains leads to morality rates approaching 100%, whereas isolates of moderate to low virulence induce a prolonged chronic disease [2]. The Shimen strain of CSFV, a highly virulent pathotype, produces a consistent clinical outcome in swine, providing a reliable method for studying viral mechanisms underlying virulence, pathogenesis, and virus–host interactions. Hence, we have used CSFV Shimen strain to study the response of PAMs upon the infection.

RIG-I, MDA5 and Laboratory of genetics and physiology 2 (LGP2) are the prototypical member of the RIG-I-like receptors (RLRs) family. Host recognition of viral infection through these Pattern recognition receptors (PRRs) normally initiate signaling pathways that can lead to the activation of transcription factors NF-kB and the IRF-3 and IRF-7 [3], which often culminate in the induction of an array of antiviral cytokines, including type I IFN [4-6] and proinflammatory cytokines such as TNF, IL-6 and IL-8 [7,8]. Studies have shown that over-expression of inflammatory cytokines considered responsible for the disorders observed during the course of the disease [9], and the rapid production of IFN-α/β are important for antiviral and autoimmune responses, leading to induced expression of hundreds of interferon-stimulated genes (ISGs) whose products direct antiviral and immunomodulatory actions that can defense against viral infections [10,11]. Therefore, the analysis of the signaling pathways that are involved in viral host defense is critical for the development of future therapeutic strategies.

Current studies have reported the roles of RIG-I and MDA5 in triggering innate defenses against RNA viruses [12,13]. Using attenuated Thiverval strain and wild-type GXW-07 isolates, our previous study suggested that CSFV infection could suppress the maturation and modulate the functions of Monocyte-derived dendritic cells (Mo-DCs) [14], and it also demonstrated that CSFV had no effect on the NF-κB signaling pathway *in vivo* and *in vitro*[15]. Although activity of NF-κB pathway have been investigated by our team, so far little is known as to how the signaling cascades linking RIG-I and/or MDA5 may be linked with IFNs induction and inflammatory cytokine expression. Therefore, in this study, we investigated the roles of RIG-I and MDA5 in CSFV infection *in vitro* and examined activities of IFNs and inflammatory cytokines in order to elucidate the mechanism of the virus infection in host, the results demonstrated that both RIG-I and MDA5 were essential and sufficient for the activation of transcription factors IRF-3 and NF-κB which induced the normal antiviral and inflammatory responses to CSFV. So, the observed findings might help to explain the immunological and pathological changes characteristically associated with infection of pigs with CSFV Shimen strain, providing important information for better understanding a potential mechanism of CSFV Shimen strain infection.

## Results

### CSFV infection causes up-regulation of RIG-I, MDA5 and IPS-1 in Porcine alveolar macrophages

**Figure 1:**
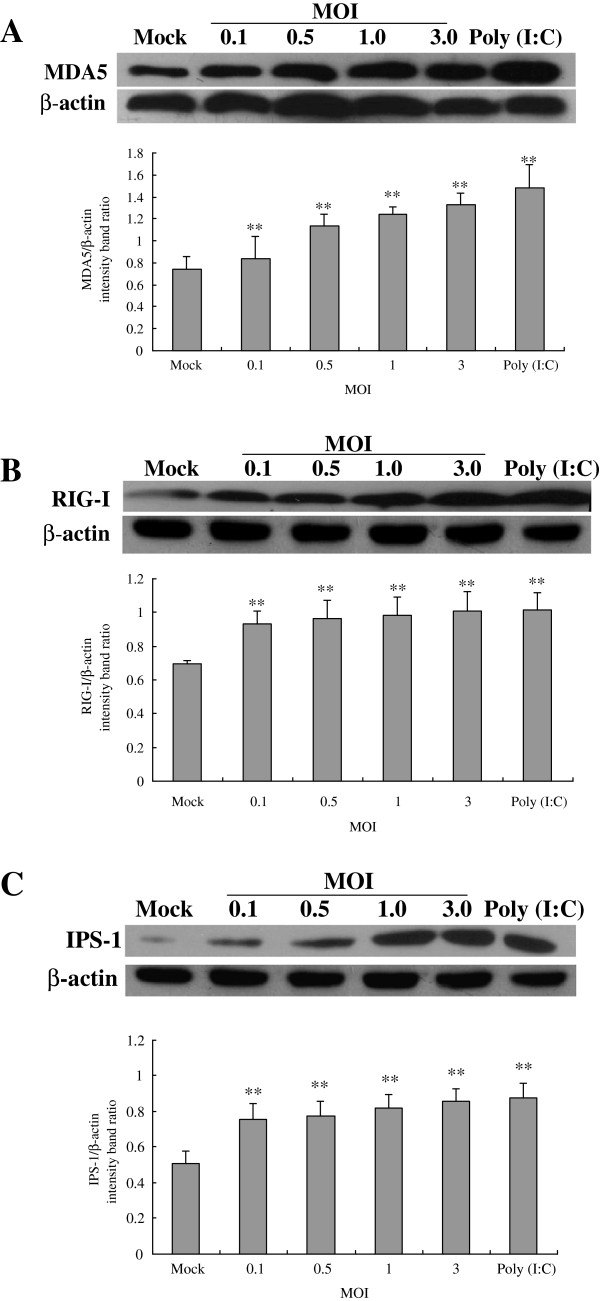
**Expression of MDA5, RIG-I and IPS-1 in CSFV-infected porcine alveolar macrophages.** CSFV Shimen isolates at MOI of 0, 0.1, 0.5, 1 or 3 were used to infect PAMs. Cells treated with poly (I:C) were used as a positive control. At 24 hpi, extracts of circa 20 μg total cell were prepared and subjected to Western Blotting for measuring protein expression of MDA-5 **(A)**, RIG-I **(B)** or IPS-1 **(C)**, respectively. Anti-β-actin was served as an internal control. The experiment was repeated three times and the figure here shows a representative experiment. An asterisk indicates a statistically significant difference from uninfected cells, * *P* < 0.05 and ** *P* < 0.01.

Following CSFV Shimen isolate challenge, Western blot analyses for the presence of RIG-1 and MDA5 associated with RIG-I signaling in PAMs were performed. The results were shown in Figure 1. Compared to the control, a higher expression of MDA5 (Figure 1A) and RIG-I (Figure 1B) was appeared in CSFV infected PAMs at 24 hpi, and the effect was dose dependent. The level of β-actin did not alter so much in all conditions, indicating no significant difference among the respective samples. Our results suggested that the CSFV infection could up-regulate the expression of RIG-I and MDA5.

To further determine whether RIG-I or MDA5 is functional to activate the RIG-I signaling pathway to trigger its downstream in CSFV-infected PAMs, we have investigated the expression of IPS-1, a critical downstream effector molecule in RIG-I signaling. As shown in Figure 1C, uninfected cells constitutively express low levels of IPS-1 but CSFV challenge significantly elevates IPS-1 expression at any MOI used. It shows that CSFV infection could activate the RIG-I signaling pathway to trigger the production of IPS-1.

### CSFV infection improves secretion of IFNs and inflammatory cytokines at different MOIs in Porcine alveolar macrophages

**Figure 2:**
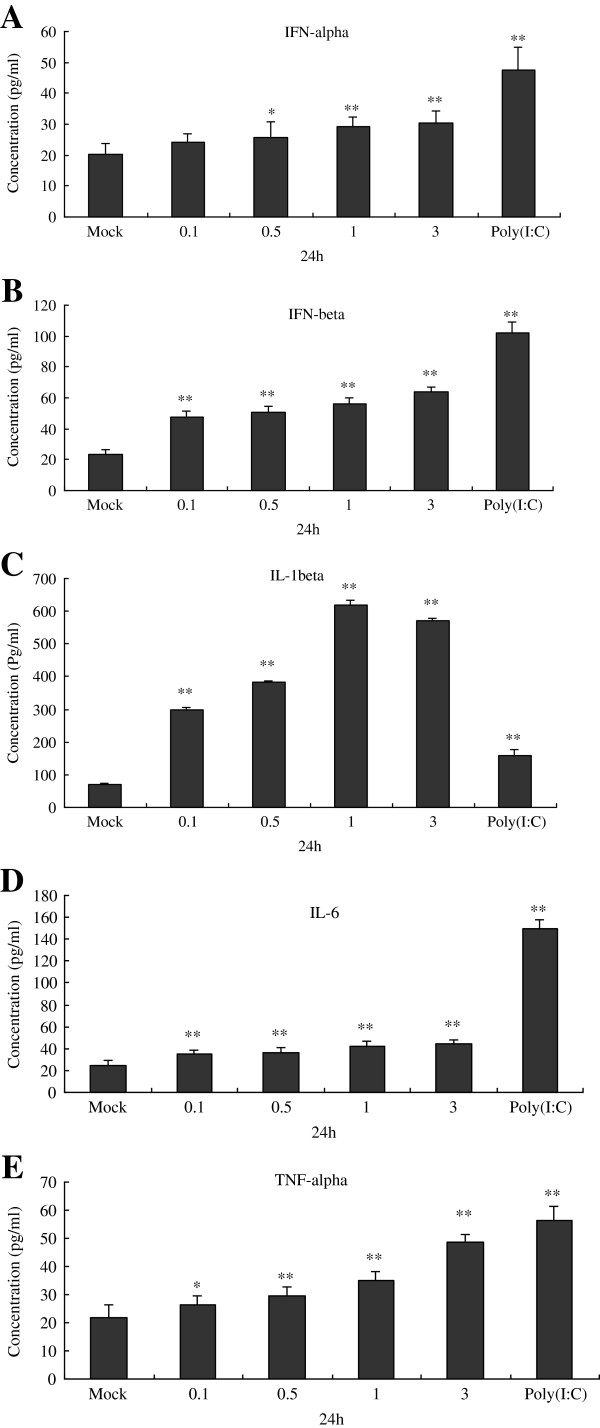
**Protein expression of IFN-α,IFN-β,IL-1β,IL-6 and TNF-α was measured by ELISA within 24 hpi following challenge of CSFV in porcine alveolar macrophages.** Cells were treated CSFV Shimen isolates at MOI of 0, 0.1, 0.5, 1 or 3. At 24 hpi, cell culture supernatants were collected to analyze the protein expression of IFN-α **(A)** , IFN-β **(B)** , IL-1β **(C)** , IL-6 **(D)** and TNF-α **(E)** by ELISA. The experiment was repeated three times and the figure shows a representative experiment. Data is expressed as mean ± SD. An asterisk indicates a statistically significant difference from uninfected cells, * *P* < 0.05 and ** *P* < 0.01.

At 24 hpi, we further examined the impact of CSFV replication on endogenous antiviral and inflammatory cytokines using ELISA. PAMs were mock infected or infected with CSFV at MOI of 0.1, 0.5, 1 and 3, and poly (I:C) was used as a positive control. ELISA analysis was performed to determine the secretion of IFN-α, IFN-β, IL-1β, IL-6 and TNF-α, the results were shown in Figure 2. It demonstrated that, as a positive control, 100 μg /ml poly(I:C) could significantly stimulate the secretion of IFN-α, IFN-β, IL-1β, IL-6 and TNF-α. Furthermore, compared to the negative control (uninfected cells), CSFV at MOI of 0.1, 0.5, 1 or 3 could promote IFN-α (Figure 2A), IFN-β (Figure 2B), IL-1β (Figure 2C), IL-6 (Figure 2D) secretion heavily, and the effect was dose-dependent. The amount of TNF-α protein in culture supernatants of infected cultures harvested at 24 h incubation (Figure 2E).

### CSFV infection promotes expression and nuclear translocation of IRF-3

**Figure 3:**
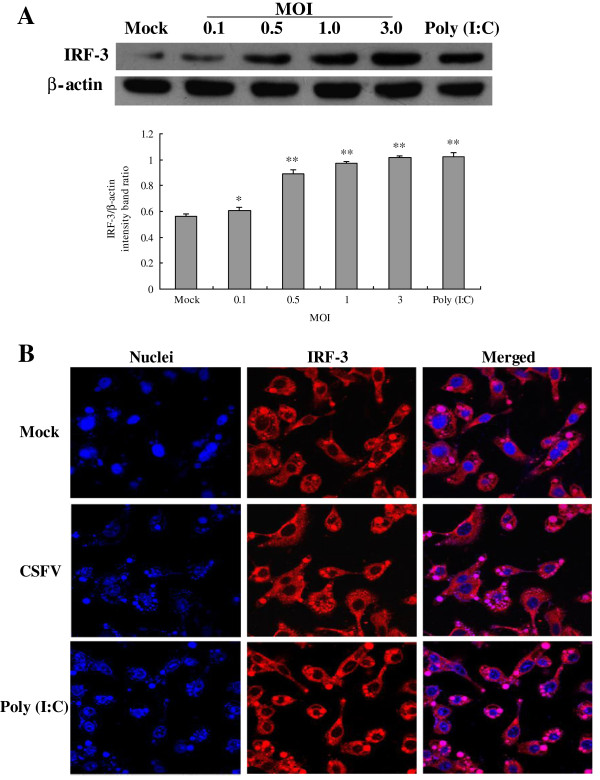
**Expression and nuclear translocation of IRF-3 after CSFV infection in porcine alveolar macrophages. (A)** Expression of IRF-3 was measured by Western Blotting with antibodies specific for IRF-3, and the cells were treated as demonstrated in Figure 1. **(B)** Indirect immunofluorescence analysis was used to measure cellular localization of IRF-3 in CSFV-infected PAMs. Cells were mock treated, poly (I:C) stimulated, or infected with Shimen isolates at an MOI of 3. At 24 hpi, cells were fixed and the localization of IRF-3 (red) was observed by fluorescence microscope using immunofluorescence stain with anti-IRF-3 and QDs-SA 605-conjugated and biotinylated secondary antibodies. Nuclei were stained with DAPI. Bar, 10 μm. The experiment was repeated three times and the figure shows a representative experiment. An asterisk indicates a statistically significant difference from uninfected cells, * *P* < 0.05 and ** *P* < 0.01.

We analyzed IRF-3 protein levels in CSFV infected PAMs by Western Blotting at 24 hpi. We found that IRF-3 protein was low in uninfected PAMs, but was significantly induced by CSFV infection (Figure 3A). The cellular localization of IRF-3 was also investigated in PAMs using immunofluorescent staining. As illustrated in Figure 3B, the localization of IRF-3 (red) was predominantly located in the cytoplasm in mock treated PAMs (Figure 3B, top panel). Conversely, the observation of nuclear staining of DAPI (blue) showed that IRF-3 was predominantly located in the nucleus in poly (I:C) stimulated cells at 24 hpi (Figure 3B, lower panel). In CSFV-infected cells, virus infection robustly induced IRF3 nuclear translocation (Figure 3B, middle panel). The results above indicate that infection with CSFV induces IRF-3 activation in PAMs.

### CSFV infection leads to activation of NF-κB/p65 in Porcine alveolar macrophages

**Figure 4:**
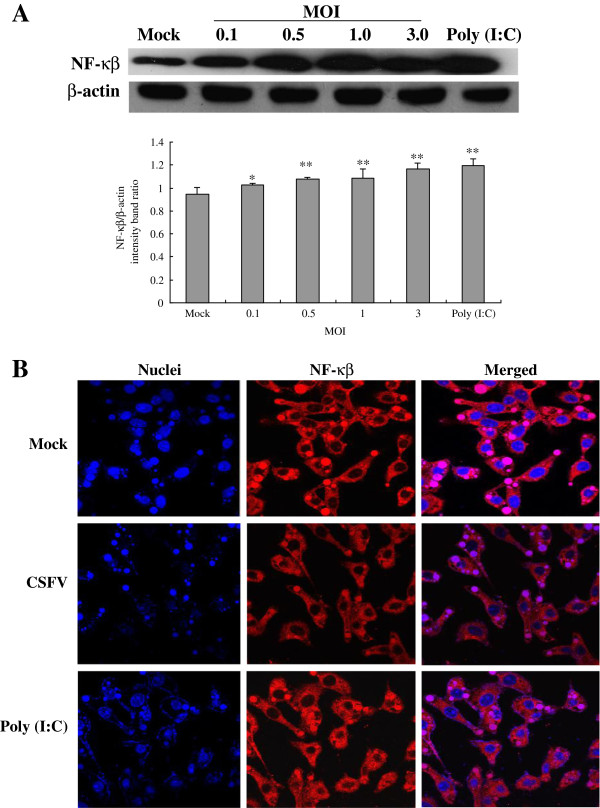
**Protein expression and nuclear translocation of NF-κB after CSFV infection in porcine alveolar macrophages. (A)** Expression of NF-κB was measured by Western Blotting with antibodies specific for NF-κB, the cells were treated as demonstrated in Figure 1. **(B)** Indirect immunofluorescence analysis was used to measure cellular localization of NF-κB in CSFV-infected PAMs. Cells were treated as indicated in Figure 3. At 24 hpi, cells were fixed and the localization of NF-κB (red) was observed by fluorescence microscope using immunofluorescence stain with anti-NF-κB/p65 and QDs-SA 605-conjugated biotinylated secondary antibodies. Nuclei were stained with DAPI. Bar, 10 μm. Representative results are shown of one of three separate experiments. An asterisk indicates a statistically significant difference from uninfected cells, * *P* < 0.05 and ** *P* < 0.01.

Protein expression and cellular localization of NF-κB were investigated in CSFV-infected PAMs, the results were shown in Figure 4. Western Blot analysis showed that CSFV could stimulate the expression of NF-κB protein in a dose-dependent manner. Notably, CSFV-induced NF-κB expression in PAMs became markedly augmented at MOI of 0.1 after infection and remained elevated at MOI of 3 (Figure 4A).

In control experiments, uninfected cells failed to signal NF-κB nuclear translocation, showing typical cytoplasmic staining of NF-κB (Figure 4B, top panel). However, in CSFV-infected experiments, we found that CSFV triggered NF-κB nuclear accumulation in a high frequency (Figure 4B, middle panel). Additionally, nuclear accumulation of NF-κB occurred within a larger frequency when cells were stimulated by poly (I:C) for 24 h (Figure 4B, lower panel). The results above indicate that infection with CSFV generates an effective signal for NF-κB nuclear translocation.

### Both RIG-I and MDA5 are required for CSFV-induced secretion of IFNs and inflammatory cytokines following CSFV infection in Porcine alveolar macrophages

**Figure 5:**
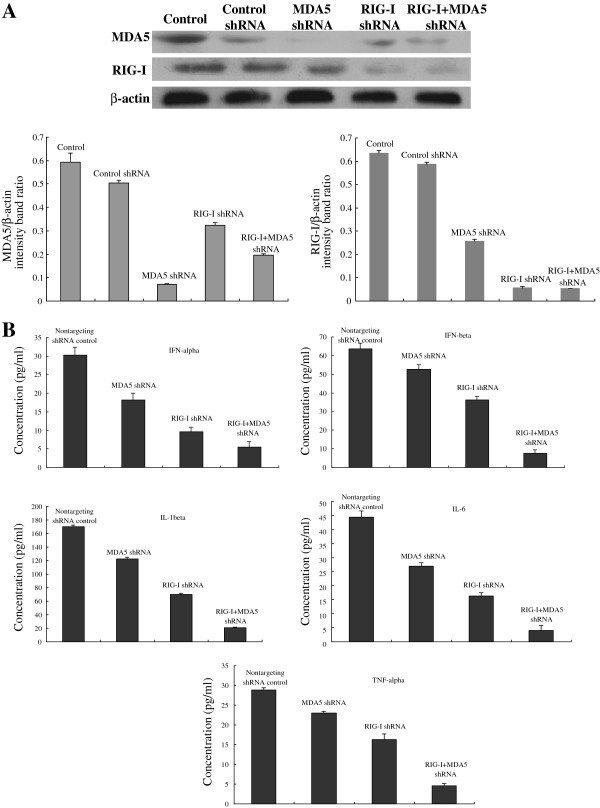
**RIG-I or/and MDA5 knockdown attenuated CSFV-induced IFN and inflammatory cytokine production by porcine alveolar macrophages. (A)** To determine knockdown efficiency, cells were untreated or transfected with shRNA targeting RIG-I or MDA5, or a combination of RIG-I and MDA5, and cultured for 24 h prior to analysis for protein expression of RIG-I, MDA5 and β-actin. **(B)** Expression of cytokines after shRNA knock-down. At 24 h following transfection, cells were infected with CSFV (MOI of 3) and cytokine secretion was measured. Data is expressed as mean ± SD. An asterisk indicates a statistically significant difference from uninfected cells, * *P* < 0.05 and ** *P* < 0.01.

To further determine the individual roles of MDA5 and RIG-I in the production of IFNs and inflammatory cytokines after CSFV infection, we established PAMs constitutively expressing shRNA targeting MDA5 and/or RIG-I. As shown in Figure 5A, following RIG-I or/and MDA5 inhibition, expression of MDA5 and RIG-I was significantly decreased. As illustrated in the section above, CSFV infection robustly induced secretion of IFNs and inflammatory cytokines at different time points in PAMs. However, the induction of IFNs and inflammatory cytokines was inhibited by knockdown of either RIG-I or MDA5 or a combine of RIG-I and MDA5. Secretion of IFN-α, IFN-β, IL-1β, IL-6 and TNF-α in response to CSFV infection was strongly reduced in RIG-I knockdown cells while it was moderately decreased in MDA5 knockdown cells. PAMs derived from knockdown of both RIG-I and MDA5 almost failed to produce IFNs and inflammatory cytokines (Figure 5B). Together, these results show that CSFV-induced IFN and inflammatory cytokine activation is dependent on the functional integrity of upstream signaling of both RIG-I and MDA5, and the effect of RIG-I is more significant.

## Discussion

Classical swine fever (CSF) caused by CSF virus (CSFV) leads to severe economic losses in pig industry especially in developing countries, many researchers have been involved in its investigations, hoping to find a better and common strategy to control its outbreaks. However, its mechanism of pathogenesis is still uncertain. Therefore, our study was conducted to explore the responses of PAMs to CSFV Shimen strain infection, for elucidating the mechanism of CSFV infection in a subtle way. Eventually, the findings found that, through binding to both RIG-I and MDA5, CSFV could activate the RIG-I signaling pathway to induce the activation of the nuclear translocation of IRF-3 and NF-κB that leaded to the production of antiviral and inflammatory cytokines.

Previous studies have shown that, although RIG-I and MDA-5 show structural similarity, they have differential roles in the recognition of different viruses. MDA5 appears to be important for sensing infections by positive strand RNA viruses such as encephalomyocarditis virus (EMCV) and poliovirus [8,16]. RIG-I is essential for the detection of a range of negative strand RNA viruses, including Sendai virus, influenza A virus, respiratory syncytial virus (RSV), vesicular stomatitis virus(VSV), Ebolavirus and hepatitis C virus (HCV) [13,17], as well as some positive strand viruses such as the flaviviruses [18]. Interestingly, RIG-I and MDA5 both contribute to the recognition of dengue virus type 2 [19] and Measles virus (MV) [20]. Kato et al. (2006) proved that mouse fibroblasts and cDCs lacking RIG-I are defective in the production of type I IFNs and inflammatory cytokines in response to various RNA viruses [13]. CSFV is sensed by MDA-5, RIG-I and TLR3 [21]. In this study, we also found that CSFV could induce a higher expression of RIG-I and MDA5, and the absence of RIG-I and/or MDA5 decreased the IFN and inflammatory cytokine production, suggesting that both RIG-I and MDA5 were involved in the recognition of CSFV Shimen strain, and the effect of RIG-I was more significant.

Following recognition of viral RNA, RIG-I and MDA5 undergo conformational changes for signal propagation to activate downstream [16]. Signaling by MDA5 or RIG-I occurs through homotypic caspase activation and recruitment domain (CARD) interactions with IPS-1 adaptor protein [22-25], which recruits RIG-I and MDA5 to the outer membrane of the mitochondria as part of a macromolecular signaling complex that serves to activate downstream IRF, NF-κB and other transcription factors [12,16]. IRF-3 and NF-κB act as molecular sensors that respond to a wide variety of changes in the environment, including those caused by viral infection [26-28]. IRF-3 is a cytoplasmic protein in its inactive state, and the stimuli can promote it to accumulate in the nucleus, where it participates in initial IFN-α/β and IFN-stimulated gene expression [29,30]. Importantly, previous studies demonstrate that IRF3 is also important for TNF expression [31-34]. NF-κB belongs to a conserved family of proteins that form multiple homo- and heterodimers with transcriptional activity [35]. In most cell types, NF-κB dimers are retained in the cytoplasm by their interaction with specific inhibitors known as IκBs. After the degradation of the IκB subunit, NF-κB is then released and translocates to the nucleus, activating the transcription of genes involved in innate and adaptive immunity [36]. Therefore, IRF-3 and NF-κB are two important factors for inducing antiviral and inflammatory responses. The results of previous studies showed that N(pro) protein in pestiviruses appeared to be essential for the virus-mediated degradation of IRF-3 by proteasomes and thus prevented IRF-3 from activating transcription from the IFN-beta promoter [37]. CSFV has evolved to prevent type I IFN sensitization in infected cells requiring both regulated dsRNA levels and the presence of viral Npro [38,39]. However, in another study, N(pro) was not degraded as a direct consequence of its ability to interact with IRF-3 or to target IRF-3 for proteasomal degradation [40]. Furthermore, CSFV N(pro) was capable of manipulating the function of IRF-7 in plasmacytoid DC [41]. In our previous studies, we ever demonstrated that attenuated Thiverval strain and wild-type GXW-07 isolates had no effect on the NF-κB signaling pathway [14]. Moreover, in present study we found that high virulent CSFV Shimen strain could activate the IRF-3 and NF-κB signaling pathway. So, we suppose the activation of macrophages via the RIG-I pathway and the resulting cytokine production contribute to the strong pathology induced by this strain.

Signaling induced by virus could trigger type I IFN production and proinflammatory cytokine expression to orchestrate the immune response against the pathogen [16]. Type I IFN, including multiple forms of IFN-α and single forms of IFN-b, IFN-u, etc., play central roles in antiviral responses [16] and also involved in the modulation of inflammation [42]. Inflammation is a protective response by the body to ensure removal of detrimental stimuli, as well as a healing process for repairing damaged tissue. Moderate inflammatory responses are advantaged to the damaged tissues to repair, and also critical for the pathogenesis of autoimmune diseases [43]. Recent studies have demonstrated that the highly active proinflammatory cytokine IL-1β is essential in antiviral host defense. Despite its essential role in host defense, high levels of IL-1β are also responsible for unwanted effects like fever, vasodilatation, hypotension or acute lung injury by fluid accumulation in response to viral infection [9]. TNF-a, IL-1a and IL-6 are three proinflammatory cytokines that form part of a complex defence network that protects the host against inflammatory agents, microbial invasion and injury [44]. However, overproduction or aberrant regulation of these cytokines may harm the host, by inducing tissue injury or alteration of the immune system [44]. As previously reported, CSFV infected macrophages and monocytes during acute infection, and macrophages sustained *in vivo* replication of CSFV [45,46]. Pigs were inoculated with CSFV and euthanized at 3, 5, 7, and 10 days post-inoculation. An increase in TNF-α mRNA expression was detected in CSFV-infected lymph nodes, and TNF-α protein was detected in lymph nodes by immunohistochemistry [47]. As indicated in the results, the production of IFN-α, IFN-β, IL-1β, IL-6 and TNF-α of PAMs is improved highly after CSFV Shimen strain infection, supposing the cells might use this way to defense the virus infection and to protect the host from harm.

## Conclusions

**Figure 6:**
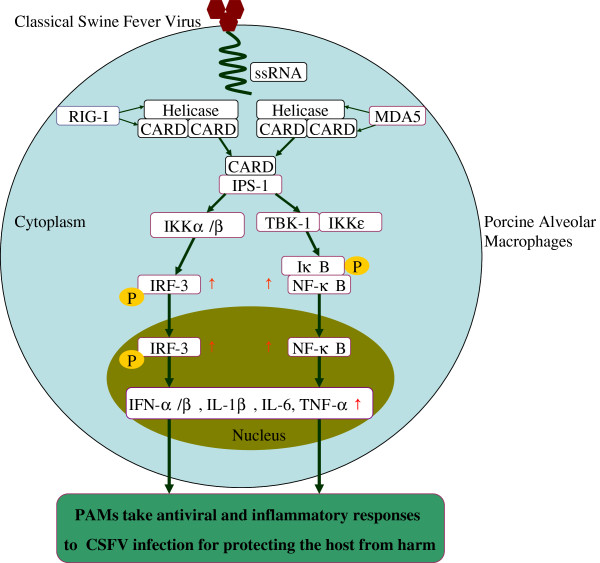
**Schematic diagram showing CSFV triggers RIG-I and MDA5 dependent signaling pathway to IRF-3 and NF-κB activation to promote the production of antiviral and inflammatory cytokines in porcine alveolar macrophages.** Detection of CSFV Shimen strain by RIG-I and MDA5 activated a cascade of changes that lead to the higher protein expression of IPS-1, stimulated the nuclei accumulation of IRF-3 and NF-κB. As a result of such activation, antiviral factors IFN-α and IFN-β were apparently promoted, and inflammatory cytokines such as TNF-α, IL-6 and IL-β were heavily enhanced in PAMs following CSFV Shimen strain infection. Ablation of RIG-I and/or using shRNA resulted in negative modulation of the secretion of antiviral and inflammatory cytokines. ↑- up-regulation.

In summary, as illustrated in Figure 6, we identify that, in CSFV-infected PAMs, MDA5 and RIG-I are both involved in virus recognition to activate the RIG-I signaling pathway for the activation of IRF-3 and NF-κB which are essential to induce the production of antiviral and proinflammatory cytokines. Understanding the role of the RIG-I signaling pathway following CSFV Shimen strain infection would contribute the important information to the molecular pathogenesis of this virus infection.

## Materials and methods

### Cells and virus

Porcine alveolar macrophage (PAM) cell line, were preserved and propagated in our laboratory (Laboratory of Microbiology and Immunology, College of Veterinary, South China Agricultural University). The cells were maintained in RPMI 1640 supplemented with 10% (vol/vol) fetal bovine serum (FBS), penicillin (100 units/ml), and streptomycin (100 mg/ml). All cells were cultured at 37°C in a humidified 5% CO2 incubator. CSFV Shimen strain used in this study was originally obtained from the blood of a pig with naturally occurring CSF and propagated on PK-15 cells. Virus titres were determined and calculated as described previously [48].

### Short hairpin RNA (shRNA)-Mediated RIG-I and/or MDA5 Knockdown

RIG-I (EU126659.1) or MDA5 (EU006039.1) knockout PAMs were generated by infecting pLLU2G-shRNA that expressed a RIG-1 or MDA5-targeting set of short hairpin RNAs (shRNAs). Briefly, PAMs were transfected using Lip2000 transfer reagent (Introgen, USA) with 50 ng nontargeting control, MDA5 or RIG-I shRNA plasmid or a combination of MDA5 and RIG-I shRNA plasmid according to the manufacturer’s recommendations. Antibiotic-free media was replaced with complete media at 6 h following transfection. At 24 h after transfection, cultured cells were infected with CSFV at a multiplicity of infection (MOI) of 3. Then at 24 hpi, whole cell lysates were collected for Western blot analyses to confirm RIG-I or MDA5 expression knockdown, and cell supernatants were collected for analyzing the expression of cytokines. The following shRNAs were used for silencing MDA5 or RIG-I expression in PAMs in this study. MDA5, 5′-TGCAGACGAAGTTTGCTGACTATCAACTCGAGTTGATAGTCAGCAAACTTCGTCTGCTTTTTCTCGAGAAAAAGCAGACGAAGTTTGCTGACTATCAACTCGAGTTGATAGTCAGCAAACTTCGTCTGCA-3′. RIG-1, 5′-TCACCAGCAAACAGCATCCTATAATCTCGAGATTATAAGGATGCTGTTTGCTGGTGTTTTTCTCGAGAAAAACACCAGCAAACAGCATCCTTATAATCTCGAGATTATAAGGATGCTGTTTGCTGGTGA-3′.

As a negative control, cells were transfected with pLLU2G-shRNA particles that expressed a nontargeting control shRNA. The shRNA used as a nontargeting control in this study was 5′-TGCGCGCTTTGTAGGATTCGCTCGAGCGAATCCTACAAAGCGCGCTTTTTCTCGAGAAAAAGCGCGCTTTGTAGGATTCGCTCGAGCGAATCCTACAAAGCGCGCA-3′. All shRNAs were purchased from Cyagen Company in China.

### Western blot analysis

For Western blot analysis, six-well dishes of cells infected with CSFV at MOI of 0, 0.1, 0.5, 1 or 3, or treated with 100 μg/ml poly (I:C) for indicated time periods. Protein extracts were prepared from cells by suspension in lysis buffer containing 0.15 M NaCl, 0.05 M Tris–HCl, 1% Triton-X 100, 0.5% sodium deoxycholate, 0.1% SDS, 1 mM sodium orthovanadate and protease inhibitor cocktail for 30 minutes on ice. Circa 20 μg of proteins were separated by 10% sodium dodecyl sulfate-polyacrylamide gel electrophoresis (SDS-PAGE) and transferred to PVDF membranes. The membranes were blocked in 5% non-fat milk for 2 hour at room temperature and incubated overnight with primary antibodies at 4°C. Following overnight incubation, the membranes were washed three times for 10 minutes with phosphate-buffered saline (PBS) containing 0.5% Tween-20 and incubated with secondary antibody at 37°C for 2 h. The membranes were then developed with enhanced chemiluminescence (ECL) substrate (Beyotime, China) and exposed to X-ray film. As a control, gels were stripped and re-probed with antibodies against β-actin. A rabbit monoclonal antibody directed against MDA5 (1/1000, Sigma, USA), a rabbit monoclonal antibody directed against RIG-I (1/1000, Imgenex, USA), a rabbit polyclonal antibody directed against NF-κB/p65 (1/1000, Thermo, USA), a rabbit polyclonal antibody directed against IRF3 (1/5000, AVIVA, China), a goat monoclonal antibody directed against β-actin (1/1000, Beyotime, China), a HRP-conjugated anti-goat secondary antibody (1/1000, Beyotime, China) and a HRP-conjugated anti-rabbit secondary antibody (1/100000, Bioworld, USA) were used in this study. Band density was quantitated using Image J software.

### Immunofluorescent staining

Subcellular localization of IRF3 and NF-κB was determined using immunofluorescencent staining. Cultured PAMs were seeded in 12-well chamber slides and either mock-infected or infected with virus at MOI of 3. Cells treated with 100 μg/ml poly (I:C) (Sigma, USA) were used as a positive control. After 24 h of incubation, cells were fixed in 80% (vol/vol) acetone for 10 min, and permeabilized for internal staining with PBS containing 0.1% Triton X-100 (Sigma, USA) for 30 min. Cells were then blocked in PBS containing 3% BSA (Sigma, USA) for 30 min and incubated with rabbit polyclonal anti-NF-κB (1:100; Thermo, USA) or anti-IRF3 (1:200, Beijing AVIVA System Biology, China) antibodies at 4°C for overnight. After washed three times with PBST, cells were further incubated with QDs-SA-conjugated and biotinylated secondary antibodies (1:100, Molecular Probes, Jiayuan Quantum Dots, Wuhai, China) at 37°C for 30 min. Cells were then mounted with 4′-6-diamidino-2-phenylindole (DAPI) for 10 min. After final washes, the coverslips were mounted onto microscope slides, and cells were visualized by a confocal laser scanning microscope (Zeiss, Germany) with the appropriate barrier filters in the Guangdong Husbandry Research Institute.

### Quantification of IFN and inflammatory cytokine expression in cell cultured supernatants

PAMs were seeded in six-well plates one day prior to virus infection. Then cell monolayers were infected with CSFV Shimen strain at MOI of 0, 0.1, 0.5, 1 or 3, respectively. As a positive control, the cells were treated with 100 μg/ml poly (I:C) (Sigma, USA). At the indicated times post-infection, cell culture supernatants were collected and used to analyze the production of IFN-α, IFN-β, IL-1β, IL-6 and TNF-α protein using enzyme-linked immunosorbent assays (ELISAs) according to manufacturer's protocols. All these ELISA kits were purchased from Uscn Life Science Inc in China.

### Statistical analysis

Results of the present study were analyzed by one-way analysis of variance and by Student’s t test with Bonferroni correction. All numerical data were collected from at least three separate experiments. Results were expressed as means ± standard deviation of the means. Results were considered statistically significant when a P value of less than 0.05 was obtained.

## Abbreviations

RIG-I: RNA helicases retinoic acid-inducible gene I; MDA-5: Melanoma differentiation-associated gene 5; CSF: Classical swine fever; CSFV: CSF virus; PAMs: Porcine alveolar macrophages; IPS-1: Interferon promoter-stimulating factor 1; IRF: Interferon regulatory factor; NF-κB: Nuclear factor-kappa B; IFN-α: IFN-alpha; IFN-β: IFN-beta; IL-1β: Interleukin-1beta; TNF-α: Tumor necrosis factor; pi: post-infection; LGP2: Laboratory of genetics and physiology 2; RLRs: RIG-I-like receptors; PRRs: Pattern recognition receptors; Mo-DCs: Monocyte-derived dendritic cells; FBS: Fetal bovine serum; shRNAs: short hairpin RNAs; MOI: Multiplicity of infection; SDS-PAGE: Sodium dodecyl sulfate-polyacrylamide gel electrophoresis; PBS: Phosphate-buffered saline; ECL: Enhanced chemiluminescence; DAPI: 4′-6-diamidino-2-phenylindole; ELISAs: Enzyme-linked immunosorbent assays; EMCV: Encephalomyocarditis virus; RSV: Respiratory syncytial virus; VSV: Vesicular stomatitis virus; HCV: Ebolavirus and hepatitis C virus; MV: Measles virus.

## Competing interest

The authors declare that they have no competing interest.

## Authors’ contributions

XYD, JJP and WJL contributed equally to this work; they designed this study, carried out ELISA analysis and drafted the manuscript; MQZ undertook CSFV Shimen strain infection and participated in the interpretation of data; JJP and JYW carried out Western Blot analysis; YWL and CMJ cultured PAMs and performed immunofluorescent staining analysis; as the corresponding author of this study, JDC participated in the design of the study and revised the manuscript. All authors read and approved this version to be published.

## Authors’ information

XY Dong and WJ Liu are involved in evaluation of immune responses after vaccination in virus-infected animals, and they also work on cell signaling pathway induced by viral infection; MQ Zhao studies transmission of CSFV and vaccine responses after viral infection; JY Wang and JJ Pei make the investigation into cell apoptosis and autophagy after viral and bacterial infection; YW Luo and CM Ju have a PhD in Preventive Veterinary Medicine and investigate pig diseases; JD Chen holds a PhD in Preventive Veterinary Medicine and works on immunology and cell signaling pathway after viral infection.
