# Correction: Classical swine fever virus triggers RIG-I and MDA5-dependent signaling pathway to IRF-3 and NF-κB activation to promote secretion of interferon and inflammatory cytokines in porcine alveolar macrophages

**DOI:** 10.1186/s12985-024-02409-8

**Published:** 2024-06-12

**Authors:** Xiao-Ying Dong, Wen-Jun Liu, Ming-Qiu Zhao, Jia-Ying Wang, Jing-Jing Pei, Yong-Wen Luo, Chun-Mei Ju, Jin-Ding Chen

**Affiliations:** 1 College of Veterinary Medicine, South China Agricultural University, No.483 Wu Shan Road, Tian He District, Guangzhou, 510642 China; 2 College of Yingdong Agricultural Science and Engineering, Shaoguan University, Daxue Avenue, Zhenjiang District, Shaoguan, 512005 China

**Correction** **:** **Virology Journal 10, 286 (2013)**

**http://www.virologyj.com/content/10/1/286**

Following publication of the original article [1], the authors identified that the captions for Figure 3A and Figure 4A are incorrect.

For Figure 3A

The incorrect caption is: "Expression of NF-κB was measured by Western Blotting with antibodies specific for NF-κB, the cells were treated as demonstrated in Figure 1"

The correct caption is: "Expression of NF-κB was measured by Western Blotting with antibodies specific for NF-κB, the cells were the same as in Figure 1B."

For Figure 4A

The incorrect caption is: "Expression of IRF-3 was measured by Western Blotting with antibodies specific for IRF-3, and the cells were treated as demonstrated in Figure 1"

The correct caption is: "Expression of IRF-3 was measured by Western Blotting with antibodies specific for IRF-3, and the cells were the same as in Figure 1B."
